# Transudative Malignancy: Uncommon and Real

**DOI:** 10.7759/cureus.39944

**Published:** 2023-06-04

**Authors:** Laura Cardenas Ramos, Saumya Batra, Kim Kyung, Mariam Farhan

**Affiliations:** 1 Internal Medicine, St. Vincent's Medical Center, Bridgeport, USA

**Keywords:** congestive heart failure, light's criteria, malignancy, transudate, pleural effusion

## Abstract

Light’s criteria are the cornerstone to differentiate exudates from transudates. The traditional literature states that malignant pleural effusions are rarely transudative; therefore, cytology tends to be low yield and not a cost-effective decision. This case describes an 82-year-old female who developed a transudative pleural effusion despite having an underlying malignancy, highlighting the importance of integrating clinical judgment into pursuing thoracentesis with the cytological examination.

## Introduction

The accumulation of fluid in the pleural space can be related to benign or malignant conditions [1]. The stratification of the pleural fluid into an exudate or a transudate is based on fluid analysis, which varies depending on the method chosen for this purpose [1]. Light’s criteria (LC) were first published in 1972 by Dr. Richard Light, and have become the standard tool to classify pleural fluid for the past four decades [1]. These criteria describe that an effusion with any of the following features is an exudate: pleural to serum protein ratio greater than 0.5, pleural to serum lactate dehydrogenase greater than 0.6, and pleural lactate dehydrogenase greater than 2/3 of the level in the serum [1]. If the fluid meets none of these criteria, it should be considered a transudate [1].

LC's ability to distinguish between exudates and transudates was confirmed in multiple studies, showing an approximate sensitivity of 90-97% and specificity of 71-80% [1,2]. Subsequent investigations have shown that almost all exudates are identified accurately, as described by Romero-Candeira et al. [3,4].

Approximately 15-30% of transudates are misclassified as exudates. This false classification is attributed in most cases to the use of diuretic therapies. By raising the pleural fluid concentration of protein and lactate dehydrogenase, diuretic therapy reduces the accuracy of LC, leading sometimes by small margins to meet exudative standards [1].

Malignant effusions that manifest as transudates are rare. Herein, we present a case of an 82-year-old female who presented with bilateral pleural effusion and was classified as transudate by LC; however, cytology was consistent with malignancy.

This article was previously presented as an abstract at the American College of Physicians (ACP) 2022 Connecticut Chapter Scientific Meeting.

## Case presentation

An 82-year-old female with a past medical history of chronic heart failure with preserved ejection fraction (HFpEF), New York Heart Association (NYHA) class II, hypertension, stage IV chronic kidney disease (CKD), and non-insulin-dependent type 2 diabetes mellitus presented with worsening generalized weakness, shortness of breath, orthopnea, and poor appetite that started one month ago and have been getting progressively worse in the past two weeks. In the emergency department, her vital signs consisted of a blood pressure of 168/70 mmHg, heart rate of 77 beats per minute, respiratory rate of 17 respirations per minute, and oxygen saturation of 88% on room air, which improved to 92% when placed on 2 L nasal cannula. Her physical exam showed dry oral mucous membranes, normal chest expansion without the usage of accessory muscles, and regular heart rhythm without murmurs. Remarkable findings were consistent with jugular venous distention, decreased basal breath sounds (right more than left), and bilateral basal rales. Lower extremities edema was not present.

**Figure 1:**
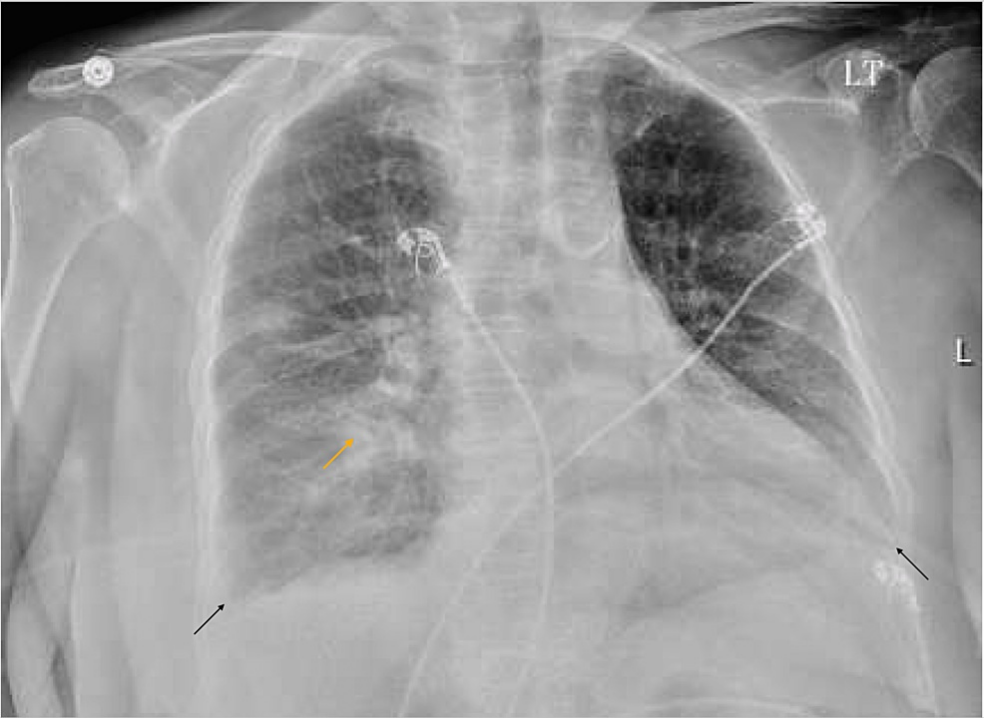
Chest X-ray on admission Mild bilateral pleural effusion (black arrow) and consolidation versus lymphadenopathy on right midlung and basilar airspace (yellow arrow).

Her laboratory assessment revealed a white blood cell (WBC) count of 14.2 T/uL (normal value: 4-11 T/uL), hemoglobin of 9.2 g/dL (normal value: 11.7-15.7g/dL), sodium of 136 mmol/L (normal value: 136-145 mmol/L), potassium of 5.4 mmol/L (normal value: 3.4-4.5 mmol/L), magnesium of 1.4 mg/dL (normal value: 1.6-2.6 mg/dL), glucose of 127 mg/dL (normal value: 74-106 mg/dL), creatinine of 2.9 mg/dL consistent with her baseline value (normal value: 0.6-1.0 mg/dL), blood urea nitrogen of 27 mg/dL (normal value: 9-23 mg/dL), glomerular filtration rate of 16 ml/min/1.73 square meters (normal value: >59 ml/min/1.73 square meters), and brain natriuretic peptide (BNP) of 2395 pg/ml (normal value: 0-99 pg/ml), with negative influenza A and B and COVID-19 tests. The chest X-ray on admission (Figure 1) revealed a right midlung opacity and mild bilateral pleural effusions. At this moment, the pocket of fluid was not big enough to perform a safe thoracentesis. Due to concerns for pneumonia, the patient was started on ceftriaxone for five days.

**Figure 2:**
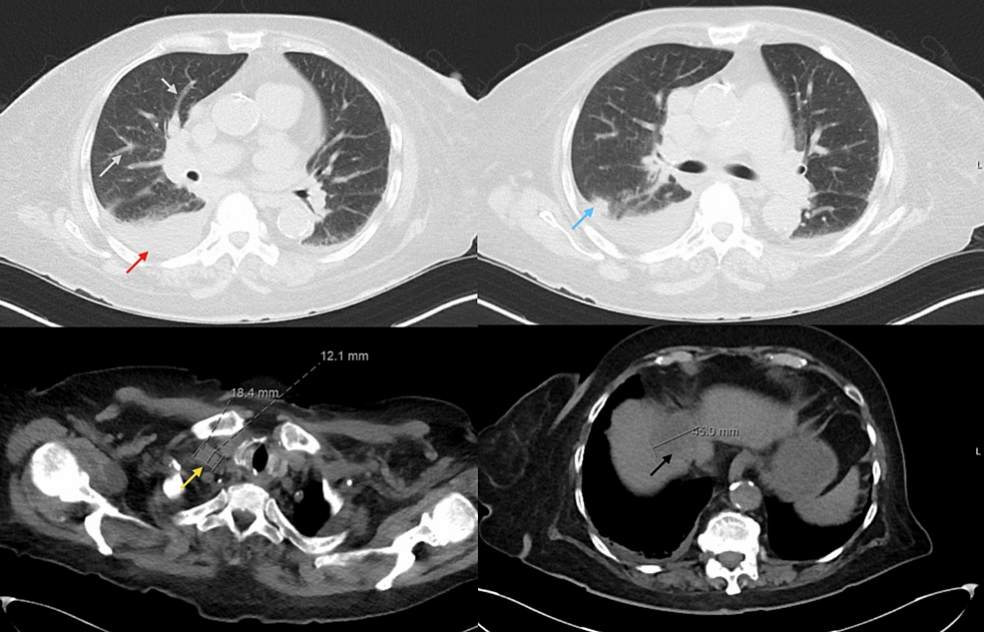
Computed tomography of the chest and abdomen without contrast Moderate right pleural effusion (red arrow), 1.4 cm ground glass nodule of the right upper lobe (blue arrow), bronchovascular thickening (grey arrow), right paratracheal lymph node (yellow arrow), and large hypodensity of the liver (black arrow).

A transthoracic echocardiogram showed an ejection fraction of 66%, with diastolic dysfunction, without wall motion abnormalities or significant valvular pathology. A computed tomography (CT) of the chest and abdomen without contrast (Figure 2) was consistent with a moderate right pleural effusion, 1.4 cm ground glass nodule of the right upper lobe, and a hypodensity of the liver (old finding), this time increased in size.

**Figure 3:**
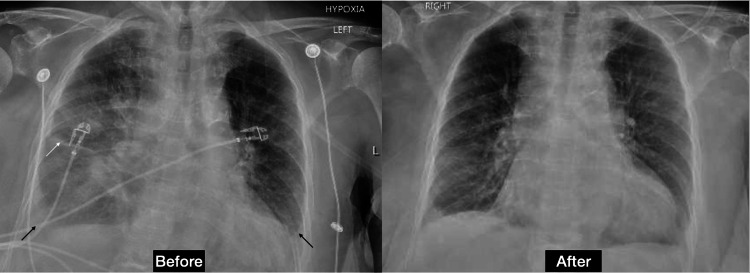
Chest X-ray before and after the first thoracentesis Worsening pleural effusion predominantly on the right side (black arrow) and Kerley line (white arrow).

Her treatment was directed toward an acute HFpEF exacerbation. She was started on intravenous furosemide, and the dose was progressively increased to 80 mg IV every eight hours and then transitioned to an intravenous drip at 20 ml/hr. Despite aggressive diuresis, on day nine of admission, she continued to be hypoxic, prompting a repeat chest X-ray (Figure 3), which showed worsening of the right side pleural effusion. A right-sided thoracentesis (Table 1) revealed 800cc of clear amber-colored fluid, with a pH of 7.52, WBC of 1394/CUMM, RBC of 2000/CUMM, neutrophils at 12%, and lymphocytes at 88%. It was classified as transudative by LC. On day 14, a CT-guided liver biopsy was performed, which reported an immunoprofile strongly suggestive of primary lung malignancy.

**Table 1:** Pleural fluid analysis Normal range: serum lactate dehydrogenase = 120-246 u/L and serum protein = 5.7-8.2 g/dL.

	1st thoracentesis			2nd thoracentesis		
	Pleural fluid	Serum fluid	Ratio	Pleural fluid	Serum fluid	Ratio
Protein	2.7 g/dL	6.2 g/dL	0.43	2.3 g/dL	5.9 g/dL	0.38
Lactate dehydrogenase	101 u/L	337 u/L	0.29	121 u/L	337 u/L	0.35

**Figure 4:**
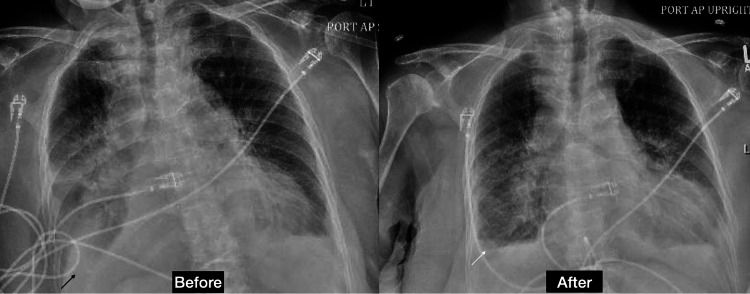
Chest X-ray before and after the second thoracentesis Moderate right pleural effusion (black arrow) and small left pleural effusion. Interval decrease of right side pleural effusion (white arrow).

A second emergent thoracentesis was done on day 16 due to severe hypoxia requiring 6 L nasal cannula. Chest X-ray findings (Figure 4) were consistent with a reaccumulation of the pleural fluid predominantly on the right side. The thoracentesis resulted to be transudative (Table 1). The pleural fluid cytology was positive for malignant cells and highly suspicious for a lung neoplasm.

**Figure 5:**
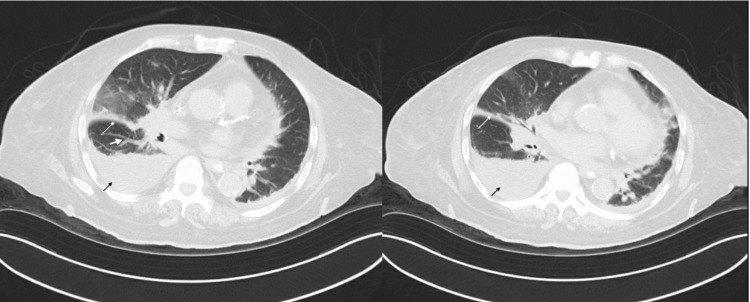
Computed tomography of the chest, abdomen, and pelvis without contrast Worsening right-side pleural effusion (black arrow) and pleural thickening along the major fissure (thin white arrow) with irregular reticular markings (thick white arrow).

A subsequent CT of the chest, abdomen, and pelvis (Figure 5) was done to rule out findings suggestive of lung lymphangitic carcinomatosis. The imaging revealed additional findings of pleural thickening along the major fissure with irregular reticular markings suggestive of lymphangitic spread. Her symptoms improved after the second thoracentesis and her oxygen requirements remained stable at 3 L. She was discharged to a short-term rehabilitation center on 3 L of supplemental oxygen per nasal cannula with oncology follow-up and an appointment for PleurX catheter placement.

## Discussion

Every new pleural effusion should be evaluated to determine if it is an exudate or a transudate, based on a standard method such as LC [5]. Transudative pleural effusions are associated with altered mechanisms that lead to the increased formation and/or decreased reabsorption of the fluid, resulting in fluid accumulation [1]. The most common pathologies associated with pleural transudates are chronic heart, renal, or liver disease [1,6]. Pleural exudates are a result of increased vascular permeability, most commonly associated with pulmonary or pleural infections and malignant processes [6]. Among ICU and non-ICU patients hospitalized with pleural effusion, 78% met the criteria for exudative fluid and 22% for transudate, and infection was described as the leading cause of exudative effusion followed by malignancy [6]. Similar results were seen in an ICU population in Egypt [7].

Pleural effusions associated with malignant processes are exudative most of the time, but it is important to keep in mind that this is not always the case. Although rare, the literature review supports that transudative malignant pleural effusion (MPE) exists. Mechanisms of transudative pleural effusion of malignant etiology have been associated with pleural seeding of the malignancy or lymphatic obstruction by the neoplasm, which can lead to an ultrafiltrate with low protein levels [8]. Light et al. described a case where the patient developed a pleural effusion in the setting of a severe heart failure exacerbation. The fluid was classified as a transudate by LC but was malignant by cytology [9]. A retrospective study by Ashchi et al. of 171 patients with known malignant disease and associated pleural effusion reported that 4.6% of the patients demonstrated a transudative effusion by LC [10]. In another retrospective review involving 122 individuals with MPE, transudative effusions were observed in 3.6% of the population [11]. A larger cohort that included 981 patients reported that 306 of this population had an MPE and 3.3% of these were transudative [12].

The most common malignancies associated with pleural effusions are lung, breast, and lymphoma. Furthermore, these malignancies are also prone to invade mediastinal lymph nodes, similar to the findings described in our case [8,10].

There are no clear recommendations regarding when it is appropriate to include cytological examination as part of the diagnostic workup of transudative pleural effusions. Ferreiro et al. studied the variables most likely associated with MPE, which included a left pleural effusion, radiological images compatible with malignancy, absence of dyspnea, and a serosanguinous appearance of the fluid [13], features that do not represent our case except for the images compatible with malignancy.

## Conclusions

Although the frequency of malignant pleural effusions manifested as transudates is low, the literature and our case lead us to encourage all physicians to integrate clinical judgment and include cytologic examination in the workup of every new unexplained unilateral or bilateral pleural effusion, especially if malignancy is suspected. By doing so, quality care and a timely diagnosis might be beneficial.
